# Differential expression of cancer associated proteins in breast milk based on age at first full term pregnancy

**DOI:** 10.1186/1471-2407-12-100

**Published:** 2012-03-21

**Authors:** Wenyi Qin, Ke Zhang, Beth Kliethermes, Rachel L Ruhlen, Eva P Browne, Kathleen F Arcaro, Edward R Sauter

**Affiliations:** 1 Department of Surgery, University of North Dakota School of Medicine and Health Sciences, 501 N. Columbia Rd., Grand Forks, ND 58202, USA; 2 Department of Pathology, University of North Dakota School of Medicine and Health Sciences, 501 N. Columbia Rd., Grand Forks, ND 58202, USA; 3 AT Still University Research Institute, 800 W. Jefferson St., Kirksville, MO 63501, USA; 4 University of Massachusetts at Amherst, 637 North Pleasant Street, Amherst, MA 01003, USA; 5 Health Sciences Department of Surgery, University of North Dakota School of Medicine, Rm 5092, Grand Forks, ND 58202, USA

**Keywords:** Lactation, Breast cancer risk, First full term pregnancy, Milk proteins Funded in part by Avon Foundation for Women

## Abstract

**Background:**

First full term pregnancy (FFTP) completed at a young age has been linked to low long term breast cancer risk, whereas late FFTP pregnancy age confers high long term risk, compared to nulliparity. Our hypothesis was that proteins linked to breast cancer would be differentially expressed in human milk collected at three time points during lactation based on age at FFTP.

**Methods:**

We analyzed breast milk from 72 lactating women. Samples were collected within 10 days of the onset of lactation (baseline-BL), two months after lactation started and during breast weaning (W). We measured 16 proteins (11 kallikreins (KLKs), basic fibroblast growth factor, YKL-40, neutrophil gelatinase-associated lipocalin and transforming growth factor (TGF) β-1 and -2) associated with breast cancer, most known to be secreted into milk.

**Results:**

During lactation there was a significant change in the expression of 14 proteins in women < 26 years old and 9 proteins in women > = 26 at FFTP. The most significant (p < .001) changes from BL to W in women divided by FFTP age (< 26 vs. > = 26) were in KLK3,6, 8, and TGFβ2 in women < 26; and KLK6, 8, and TGFβ2 in women > = 26. There was a significant increase (p = .022) in KLK8 expression from BL to W depending on FFTP age. Examination of DNA methylation in the promoter region of KLK6 revealed high levels of methylation that did not explain the observed changes in protein levels. On the other hand, KLK6 and TGFβ1 expression were significantly associated (r^2 ^= .43, p = .0050).

**Conclusions:**

The expression profile of milk proteins linked to breast cancer is influenced by age at FFTP. These proteins may play a role in future cancer risk.

## Background

Immediately following parturition there is an increased risk of breast cancer observed for all age groups [1]. This is not surprising, since the growth factors required to allow breast glandular proliferation, as well as the extensive remodeling required during involution, may stimulate the growth of already present neoplastic mammary epithelial cell(s). Over the long term, parity is protective for women whose first full term pregnancy (FFTP) was completed at a young age (variously defined as < 20 up to age 26), and increased in parous women whose FFTP occurred after 35 years of age [2]. Rodent models suggest that early FFTP leads to changes in the pattern of breast lobular development and differentiation, cell growth, and gene expression [3]. Early FFTP imprints a specific genomic signature on the breast [3]. Most studies demonstrate a protective effect of breast feeding [4], though the influence of breast feeding duration on cancer risk is less clear, with a recent large study demonstrating no effect on risk of premenopausal breast cancer [5]. We think that breast cancer associated proteins can provide clues to how early FFTP confers a protective effect on the breast.

There is good evidence that during involution, which is initiated by weaning, the breast microenvironment becomes tumor promotional [6]. Our strategy was to identify protein biomarkers in breast milk that are associated with the protective effect of early FFTP vs. the cancer promoting effect of late FFTP. We have identified cancer associated proteins in breast nipple aspirate fluid, fluid from the milk ducts of nonlactating women, including kallikrein related peptidases (KLKs) [7,8], basic fibroblast growth factor (bFGF) [9], and YKL-40 [10].

The KLK family consists of 15 highly conserved serine proteases. Several members of the family have been reported as potential cancer biomarkers [11], and many KLK genes are differentially expressed in hormone-related malignancies [12]. It has previously been demonstrated that multiple KLKs are detectable in breast milk [12]. Furthermore, the epigenetic regulation of KLK6 expression has been demonstrated in hormone sensitive (T47D) and insensitive (MDA-MB-231) malignant breast cells [13].

bFGF [14] has been detected in human milk, while YKL-40 (and its bovine homologue, mammary gland protein (MGP)-40) levels in breast milk rise during the time of breast involution, as the breast returns to the prepregnancy state [15]. Neutrophil gelatinase-associated lipocalin (NGAL, a.k.a. lipocalin 2) is a small secreted glycoprotein which binds matrix metalloproteinase-9 to protect it from degradation [16]. NGAL is expressed in breast carcinomas, inhibition of NGAL impairs breast tumorigenesis and metastasis [17], and increased NGAL expression has been associated with decreased disease-specific survival [18]. The homolog of NGAL has been identified in cow's milk [19].

Transforming growth factor (TGF)β is linked to breast cancer in preclinical models [20] and to prognosis in human breast cancer [21]. In mouse models, TGFβ is induced during mammary gland lactation and involution [20], and it mediates proapoptotic effects during involution [1]. Transgenic mice that overexpress TGFβ show increased apoptosis in the mammary epithelium throughout mammary development [22]. KLK6 is thought to act on the TGFβ1 signal transduction pathway and thereby influence cell migration and motility, and KLK6 alters the expression of TGFβ1 in breast cancer cells [23].

The time of weaning, a period of breast involution and remodeling, appears critical to future breast cancer risk [6]. The analysis of wean milk will provide essential information regarding the biology of markers related to breast involution. We investigated 16 proteins: 11 KLKs, bFGF, YKL-40, as well as NGAL and two isoforms TGFβ, which, like bFGF and YKL-40, have been associated with breast cancer [1,18]. This study was conducted based on the hypothesis that lactation exerts a protective effect by upregulating cancer preventing and downregulating cancer promoting proteins in the breast based on age at FFTP.

## Methods

### Recruitment

Healthy women were prospectively recruited prior to or soon after delivery. They were eligible if they delivered a ≥ 37 weeks gestation infant and were planning on breast feeding. After agreeing to enroll in an Institutional Review Board approved project, three milk samples were requested from each participant: baseline (BL), defined as within 10 days of the initiation of lactation, two months after lactation started, and when the woman is weaning (W). The participant was asked to provide the wean sample once they had decided to stop nursing, and had started to decrease the number of daily feedings. At least two samples from a given woman were required in order for the samples to be included in our analyses. Each sample was collected from the same breast.

Subjects were recruited in Columbia, MO, Grand Forks and Fargo, ND, after IRB approval. Mothers were asked not to breast feed their infants for at least two hours before milk collection, which involves draining the breast using manual extraction or a breast pump. Samples were immediately frozen after collection. Collected milk was thawed, centrifuged (1500 × g, 20 min, 4°C), and the fat and cellular layers separated. The aqueous phase was then centrifuged at 12000 g for 15 min at 4°C, the second lipid layer removed and stored at -80°C prior to analysis.

### Assessment of protein biomarkers

#### Total protein

25 μL of standard and milk samples were added in duplicate to each microplate well (Pierce, Rockford, IL), followed by 200 μL of working reagent with mixing. The microplate was then incubated at 37^ο^C for 30 minutes and absorbance measured at 562 nm.

#### KLKs

The concentration of KLKs was measured with a highly sensitive and specific non-competitive immunoassay. Each assay incorporated two KLK specific monoclonal and or polyclonal antibodies (Ab), raised in mouse and rabbit respectively. One Ab was used for antigen capture and other for detection (Table 1) in a sequential two site immunometric format with time resolved fluorescence detection. KLK2 and KLK3 antibodies were obtained from MedixBiochemica (Kauniainen, Finland). The remaining KLK antibodies were developed in the laboratory of Dr. Eleftherios Diamandis Director of Clinical Biochemistry, University of Toronto. The KLK assays have a detection limit ranging from 0.005 μg/L for KLK3 to 0.2 μg/L for KLK7, 8 and 11 and a dynamic range of 10-20 μg/L, depending on the KLK (Table 1). These assays have been used in multiple prior publications [8,12,24-27]. Standards (recombinant KLK) and samples were analyzed in duplicate with inclusion of three quality control samples in every run.

**Table 1:** Demographics and Kallikrein Assay Information

Demographics			
Total participants (N)		72	

First Full Term Pregnancy		35	

Multipara		37	

	2^nd ^child	25	

	_3_^rd^	5	

	4^th ^or greater	7	

Age			

	Mean (median)	28.3 (28)	

	Range	20-39	

**Kallikrein (KLK) Assay Information**			

**Kallikrein**	**Assay Configuration^1^**	**Dynamic range**	**Detection Limit**

		**(μg/L)**	**(μg/L)**

KLK2	Mono-Mono	10	0.01

KLK3	Mono-Mono	10	0.005

KLK4	Mono-Poly	20	0.1

KLK5	Mono-Mono	10	0.05

KLK6	Mono-Mono	20	0.05

KLK7	Mono-Mono	20	0.2

KLK8	Mono-Mono	20	0.2

KLK10	Mono-Mono	10	0.1

KLK11	Mono-Poly	20	0.2

KLK13	Mono-Mono	10	0.1

KLK14	Mono-Poly	10	0.1

1: Assay configuration: The confirmation listed indicates the two antibodies (Ab) used, the first being the antigen capture and the second the detection antibody. Mono: monoclonal Ab, poly: polyclonal Ab

#### bFGF

bFGF was analyzed using an enzyme linked immunosorbant assay (ELISA) kit from R&D Systems (Minneapolis, MN), following the manufacturer's instructions. The kit utilizes a quantitative sandwich enzyme immunoassay technique. The detection limit of the kit is 10 ng/L.

#### YKL-40

Samples were analyzed by immunoassay as per the manufacturer's instructions (Quidel Corporation, San Diego, CA). The kit uses a monoclonal anti-YKL-40 antibody conjugated to biotin which binds to streptavidin and captures YKL-40 in the standard or sample. The detection limit is 10 ng/mL.

#### NGAL

NGAL content in milk was measured by immunoassay (BiPorto Diagnostics, Denmark) according to the manufacturer's instructions. Briefly, 100 μL of standard and diluted milk samples were added to wells coated with a monoclonal antibody against human NGAL. Bound NGAL was detected with a second antibody labeled with biotin and the signal developed with horseradish peroxidase-conjugated streptavidin and substrates, the reaction ended with stop solution and absorbance measured at 450 nm. The detection limit of the NGAL kit is 4.0 pg/mL.

#### TGFβ isoforms

The protein expression levels of TGFβ1 and β2 in milk samples were determined by immunoassay (R&D Systems) following the manufacturer's instructions. Briefly, 50 μL of standard and activated milk samples were added to wells in duplicate, incubated for 2 hours at room temperature (RT), washed and 100 μL of TGFβ1 or β2 conjugate added. Following another wash, 100 μL of substrate solution was added for 30 min at RT, the reaction ended with 100 μL of stop solution and absorbance measured at 450 nm. The detection limits of the TGFβ1 and β2 kits are 4.61 and 7.0 pg/ml, respectively.

### Assessment of KLK6 methylation

#### DNA Isolation and bisulfite treatment

DNA was isolated from all 42 breast milk cell pellets that were available from women who underwent KLK6 protein analysis women using the QIAamp DNA mini kit (Qiagen, Germantown MD) with the following modifications: 10 μl of RNAse A (1 mg/ml) was added to each DNA isolation at the beginning of the protocol; at the end of the protocol an ethanol precipitation was performed to clean and concentrate the DNA. DNA quantity and quality were assessed using the NanoDrop 8000 spectrophotometer (Thermo Scientific, Wilmington DE). Up to 500 ng of high quality DNA were bisulfite converted using the EpiTect Bisulfite Kit according to manufacturer's instructions (Qiagen, Germantown MD).

#### KLK6 PCR and pyrosequencing

Two μl of bisulfite converted DNA were used in each PCR reaction to amplify the promoter region of the KLK6 gene using the PyroMark PCR kit (Qiagen, Germantown MD). KLK6 primers were designed to amplify an area of the promoter region upstream of the transcriptional start site (TSS). This region includes 4 CpG sites (-72, - 64, -56 and -53 upstream of TSS) that have been shown to be methylated in breast cancer cell lines and unmethylated in normal cell lines [13]. Primer sequences are as follows: sense primer: 5'- GTAAAGGAGGATTGTTAGATAGGG-3', antisense primer: 5'-biotin-CCAACACCCCAATACCAT-3', sequencing primer: 5'-GATAAAAGGAAGTTATTGATG-3'. Pyrosequencing was performed using a PyroMark Q24 pyrosequencer (Qiagen, Germantown MD) [28] using PyroGold Reagents (Qiagen, Germantown MD). Pyrosequencing using mixtures of in vitro methylated and unmethylated DNA demonstrated a high degree of linearity (R^2 ^= 0.99) for this KLK6 DNA methylation assay.

### Statistical analysis

Expression levels of proteins were first logarithm-transformed using natural base prior to analysis. Changes in protein expression over three time points (BL, 2 months, W) were tested with longitudinal analysis using a linear mixed model. P values were adjusted for multiple tests with Holm's step-down procedure. For proteins that showed a significant change over the three time points, a paired *t *test was performed to detect the change between each pair of time points. Regression analysis was carried out to identify the association between the ages at FFTP with BL protein levels or change of protein expression between the three lactation periods. In order to identify the maximum number of possible associations with FFTP, we did not adjust p values for multiple tests. The effects of nursing time on total protein and individual proteins were assessed using regression analysis. Levels of KLK6 methylation were compared among the three time points using one-way analysis of variance. The association of KLK6 methylation and protein levels was tested using regression analysis at each time point and at each CpG site. The interaction of KLK6 and TGFβ1 was analyzed using regression analysis.

## Results

Breast milk samples were collected from 72 women, 35 women for whom it was their FFTP and 37 for whom it was not (Table 1). One woman enrolled in the study after breastfeeding for 2 months and no BL milk was available. A 2 month milk sample was not collected from two women because they weaned prior to the 2 month time point. Milk was collected at all three timepoints from 47 women. Wean milk samples were not collected from 25 women (14 have not yet weaned, 9 weaned but did not provide a wean sample, and 2 were lost to follow-up).

There was considerable variability between samples in total milk protein concentration. For this reason, all biomarker results are listed and all statistical analyses were conducted controlling for total protein concentration in the milk sample. Total protein expression was not influenced by either age at FFTP or whether the samples were collected from a primiparous vs. a multiparous woman. FFTP age was not found to interact with parity status.

### The expression of multiple milk proteins varies during the course of lactation

For our two milk collection time points, overall (considering all sample collections) change in individual protein expression was compared in women with young (< 26 years) vs. older (> = 26 years) age at FFTP (Table 2). Of the 16 proteins evaluated, there was an overall change in the expression of 14 (for women < 26) and 9 (for women > = 26) proteins, respectively.

**Table 2:** Mean Natural Log (ng/g) Values of Baseline (BL), 2 Month (2 Mo) and Wean (W) Samples vs.

*Age (yrs)*	*< 26*					*> = 26*				
***Biomarker***	**BL**	**2Mo**	**W**	**P (overall)**	**P (pairs)^1^**	**BL**	**2Mo**	**W**	**P (overall)**	**P (pairs)^1^**

KLK2	0.91	0.96	0.89	NS^2^	NS^2^	1.03	1.09	0.93	NS	NS

KLK3	2.36	1.75	0.80	0.0011	*, †††	2.42	1.56	1.89	NS	*

KLK4	-0.75	-0.68	0.13	0.0088	†, ^	-0.88	-0.65	-0.09	NS	††

KLK5	8.51	7.83	9.17	5.49E-11	**, ^^	8.43	7.21	8.92	1.22E-10	***,^^^

KLK6	9.88	10.1	10.9	4.61E-11	***, †††,^^^	9.79	9.89	10.6	1.56E-6	†††,^^^

KLK7	5.52	4.45	6.18	0.0039	**, ^^	5.03	4.79	5.25	NS	NS

KLK8	8.10	8.32	9.54	5.16E-10	*,†††.^^^	7.84	8.20	9.04	1.49E-10	***,†††,^^^

KLK10	5.18	5.97	7.18	0.0011	**,††,^^	4.77	5.59	7.15	2.3^-4^	*,††,^^

KLK11	6.44	4.73	6.57	5.35E-5	***,^	6.36	4.95	5.78	0.0049	**

KLK13	2.56	1.87	1.25	NS	†,^	2.11	2.01	1.14	NS	NS

KLK14	1.10	0.89	1.46	0.046	††,^^	1.18	0.83	1.21	NS	^^

**N(all KLKs)**	**37**	**36**	**20**			**32**	**33**	**21**		

YKL40	12.1	10.6	12.2	2.38E-6	***,^^	12.3	10.1	12.0	3.88E-11	***,^^^

NGAL	8.31	7.23	8.37	4.5E-4	***,^^	8.91	7.16	8.36	6.96E-12	***,^^^

bFGF	-1.46	-3.15	-1.91	2.58E-8	***,^^^	-1.07	-2.89	-1.85	NS	***,††,^^

TGFβ1	3.88	3.52	4.23	5.49E-5	**,^^	3.88	3.36	4.40	2.34E-5	**,†,^^^

TGFβ2	5.55	5.25	7.06	7.27E-7	†††,^^^	5.47	5.03	7.09	2.74E-12	*,†††,^^^

**N(non-KLKs)**	**37**	**36**	**21**			**34**	**34**	**26**		

Age at First Full Term Pregnancy

1: P (pairs): paired comparisons. *: BL vs. 2 Mo; †: BL vs. W; ^: 2 Mo. vs. W. One mark (*, †, or ^): p < 0.05; two marks: p < 0.01; three marks: p < 0.001. P values adjusted for multiple comparisons using Holm's step-down procedure

2: NS: not significant. If NS is listed under P (pairs), it indicates that there were no significant associations for any of the three paired comparisons

For proteins with a significant global change, we determined (Table 2) the change for each of three lactation periods (BL to 2 months, BL to W and 2 months to W). Mean expression significantly decreased for 8 (for women < 26) and 8 (for women > = 26) proteins, respectively, between BL and 2 months, and increased for 13 (for women < 26) and 11 (for women > = 26) proteins, respectively, between 2 months and W. Between BL and W there was a highly significant (p < .001) increase in the expression of KLK6, 8, and TGFβ2 in both age groups. KLK3 expression was lower in W than BL milk of women < 26 but not those in the > = 26 group.

### Expression (and expression change during lactation) of biomarkers linked to breast cancer are related to age at FFTP

**Figure 1:**
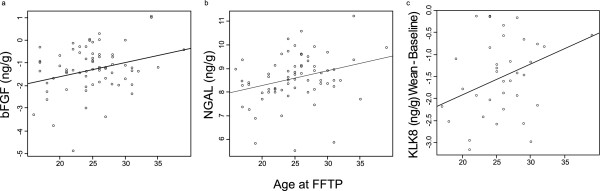
**Natural log a) bFGF and b) NGAL baseline milk expression; or c) change (wean--baseline) in natural log KLK8 milk protein expression based on age at FFTP**. For KLK8, only matched samples are represented.

For each of the 16 proteins, we evaluated the association of expression with age at FFTP. At BL, bFGF (p = .0116) and NGAL (p = .012) expression were significantly higher in women who were older at the time of their FFTP (Figure 1a,b). There were no significant associations at 2 months or W. There was a larger increase in KLK8 expression (p = .022) from BL to W (controlling for total time that a woman nursed) with younger age at FFTP (Figure 1c).

### DNA methylation of KLK6 varies among women and with breast feeding duration but does not explain protein expression pattern

**Figure 2:**
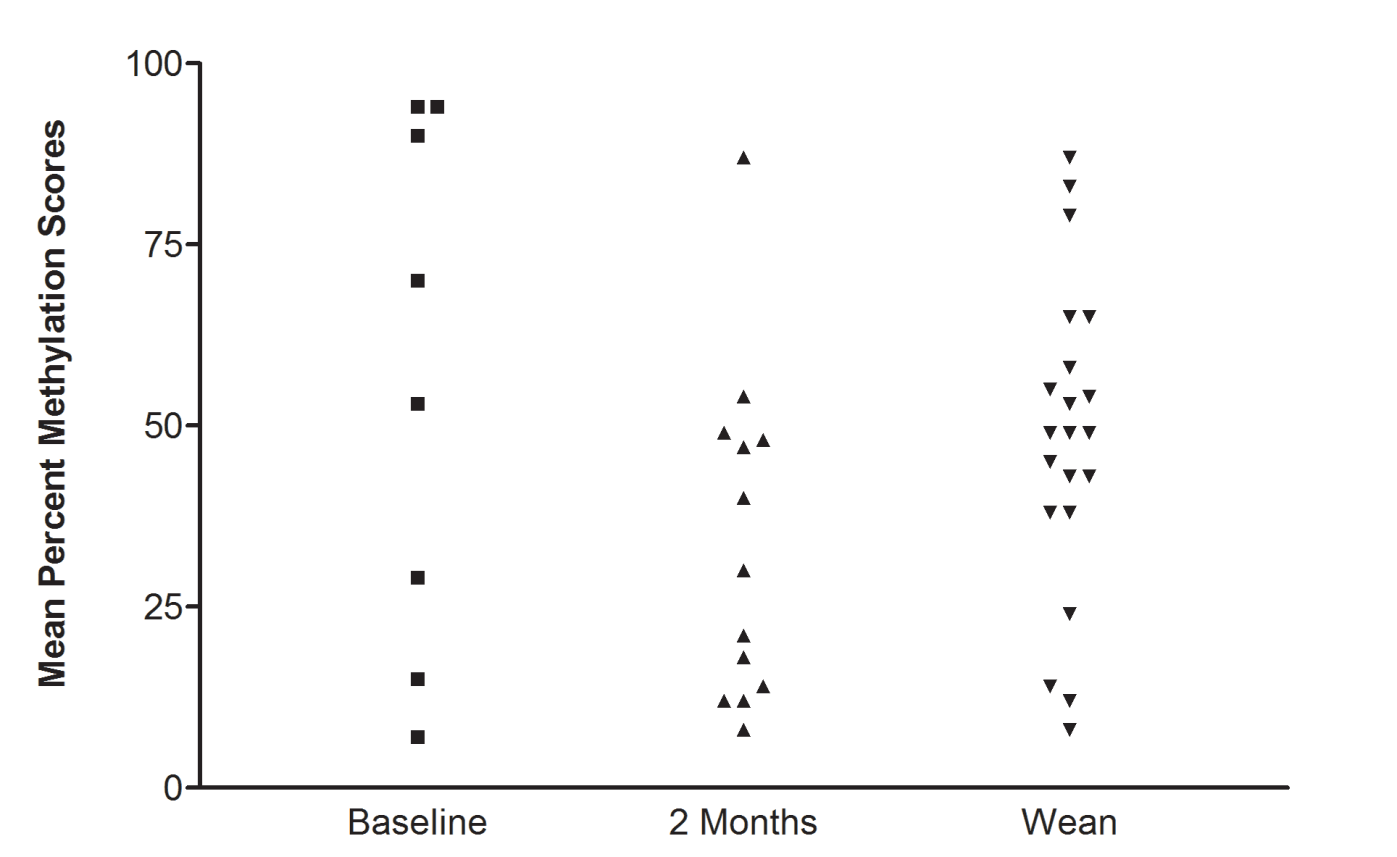
**Methylation of KLK6 in DNA isolated from cells obtained from breast milk**. The average of the percent of methylated DNA at each of 4 CpG sites in the proximal promoter of KLK6 is shown for milk samples collected at Baseline (n = 8), Two Months (n = 13), and Wean (n = 21).

To determine the extent to which the changes observed in KLK6 protein levels were controlled by epigenetc events, we isolated DNA from a total of 42 breast milk cell pellets (eight at BL, 13 at 2 months and 21 at W) obtained from 32 women. Methylation analysis by pyrosequencing revealed high mean levels of KLK6 promoter methylation at all three time points 57% (BL), 34% (2 months) and 48% (W), and no significant differences across time points (F = 2.27; p = .117). Among the ten women for whom methylation was assessed at two time points, there was an average absolute difference of 37% methylation between the two sampling times. The greatest differences (Figure 2) occurred between BL and 2 months (mean change for the 6 women = 44%) and between 2 months and W (mean change for the two women = 42%) with little change between BL and W (mean change for the two women = 10%). The methylation level was not associated with KLK6 protein level (p = .35).

### Association of KLK6 with TFGβ1

**Figure 3:**
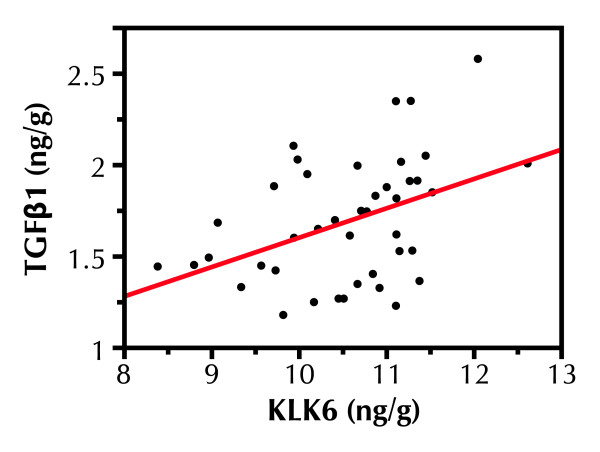
**Association of natural log TGFβ1 and KLK6 protein expression in breast milk**.

KLK6 has been reported to interact with the TGFβ1 signal transduction pathway and influence TGFβ1 expression in breast cancer cells [23]. We determined the association of these proteins in breast milk, considering all samples for which there were paired values. The two proteins (Figure 3) were significantly associated (r^2 ^= .43, p = .0050).

## Discussion

One approach to assess the influence of a specific process such as age at FFTP is global (proteome wide) assessment. Unfortunately, this approach has limited sensitivity [29] even with sample concentration approaches [30], and lack reliable quantitation of individual protein concentration. We elected to use immunoassays which are highly sensitive and quantitative, to compare individual protein expression during the course of lactation.

A striking feature of the data is that the expression of most proteins varies during lactation, even after controlling for total protein concentration. A similar observation in a smaller sample was made by another group in the assessment of milk immunoglobulins and lysozyme [31]. The most common trend that we observed was for the concentration of proteins to decrease from BL to 2 months after starting nursing, and then to increase between 2 months and W. Although the reason(s) for this are uncertain, a possible explanation is that the breast secretes a given amount of each protein daily, with greater milk volume during the 2 month lactation period compared to BL or W periods leading to a lower protein concentration. Indeed, total milk protein concentration has been shown to negatively correlate with 24 hour milk volume [32], and gradual weaning over a period of three months has been shown to increase total protein as milk volume decreased [33].

The most significant changes in expression from the start to the end of lactation in both FFTP age groups were in KLK6, 8 and TGFβ2. There was a significant decrease in KLK3 in women < 26 at the time of FFTP, but not in women with a later age of FFTP. The significance of the change in one but not the other group is uncertain. For KLK6, 8 and TGFβ2, there was a small change early (between BL and 2 months), with a larger change later (between 2 months and W) in lactation. For all three proteins there was an increase in mean expression at the time of weaning compared to BL.

KLK6 and 8 expression is downregulated in breast cancers [34]. As such, it has been speculated that these genes may function to suppress tumors [13]. KLK6 [13] is epigenetically regulated with gene silencing in tumors. KLK6 may play a protective role against tumor progression by inhibiting the epithelial-to-mesenchymal transition [13]. KLK8 has been shown to function as a serine protease [35]. The mechanism of KLK8 gene silencing in breast cancer has not been reported, though alternative splice variants of the gene which influence prognosis have been reported in lung cancer [36]. The increase in KLK8 expression from BL to W was greatest in women who whose FFTP occurred at a young age, which is consistent with known protective effect of early age at FFTP [2] and a tumor suppressive effect of KLK8.

The significant changes in KLK6 protein expression during lactation and the report of regulation of KLK6 by methylation in breast cancer cells [13] prompted us to examine KLK6 promoter methylation in cells obtained from breast milk. We detected surprisingly high levels of KLK6 promoter methylation with moderate differences among the three sampling times that did not explain the observed changes in protein expression. Indeed, the lowest mean level of methylation, 34%, occurred at 2 months. While we expected low methylation to be associated with the highest protein expression, KLK6 expression, on average, was highest in wean milk for all age categories (Table 2). Whereas methylation scores were relatively high at all sampling times, repeated measures from ten women revealed average methylation changes during lactation of 37%.

To assess the likelihood that the observed variability in *KLK6 *methylation throughout lactation was an artifact, we examined the promoter methylation of *p16 *(data not shown), which we would not predict to vary with lactation duration. In contrast to KLK6, there was little methylation of p16 at each point, ranging from 1 to 5%, indicating that the variability in KLK6 methylation is not likely to be an artifact. It is important to note that, unlike the prior report associating KLK6 methylation with KLK6 expression in cultured malignant breast cells [13], our clinical investigation evaluated breast cells collected from the breasts of healthy women. Regarding the variability in KLK6 methylation which we observed, we believe that this is at least partly due to differing percentages of epithelial cells and leucocytes that were present in the milk at the three sampling times, since DNA was isolated from the total cell pellet. Nonetheless, we confirmed that epithelial cells present in milk from healthy breasts have high levels of methylation by examining several epithelial-enriched cell fractions (data not shown).

Whereas we did not find a association between KLK6 methylation and protein expression, we did observe a highly significant association between KLK6 and TGFβ1 protein expression. TGFβs regulate normal breast development, apoptosis and matrix remodeling during breast involution induced by breast weaning, with biphasic effects on tumor progression, acting as tumor suppressors in early stages of cancer and promoting invasion and metastasis at later stages [37]. Our observation that the expression of these proteins are directly correlated, whereas they are inversely associated in breast cancer cells in culture [23], is consistent with the pleiotropic effects of these molecules to suppress cancer initiation and promote later stages of cancer invasion and metastasis.

This report has limitations. Most notable is the limited sample of women whose FFTP was age 35 or greater, as well as the limited BL and 2 month sample sizes for the methylation analyses. We may have missed associations with age at FFTP due to this. A second limitation is that there was some variability between when the wean milk sample was collected and when the woman stopped nursing entirely. A third limitation is that we cannot say with certainty that the proteins identified which are upregulated at the time of weaning would put these women at lower risk of future breast cancer, since we do not have sufficient follow-up to address this. On the other hand, as previously discussed, we know from other reports that higher expression of KLK 6, 8, 10 and TGFβ2 have been associated with a lower risk of breast cancer [34,38].

## Conclusions

We report the upregulation of KLK 6, 8 and TGFβ2 at the time of breast weaning and involution. All four proteins have a tumor suppressive effect, consistent with the known protective effect of pregnancy in younger women. KLK8 levels were generally lower at BL in women older at the time of their FFTP, but the change was greater in these women, suggesting a protective correction toward a higher KLK8 expression level by the end of lactation. bFGF and NGAL expression was higher in BL lactation samples among women with an older age of FFTP. Both are tumor promotional, consistent with the association of late FFTP with increased breast cancer risk. Finally, we observed a significant association between KLK6 and TGFβ1, which may be one mechanism by which these molecules interact during lactation to influence future breast cancer risk. We plan to investigate these proteins in the nipple aspirate fluid of women with and without breast cancer, to see if their expression is associated with disease.

## Abbreviations

BL: Baseline; bFGF: Basic fibroblast growth factor; ELISA: Enzyme linked immunosorbant assay; FFTP: First full term pregnancy; KLK: Kallikrein related peptidase; MGP: Mammary gland protein; NGAL: Neutrophil gelatinase-associated lipocalin; RT: Room temperature; TGF: Transforming growth factor; TSS: Transcriptional start site; W: Wean.

## Competing interests

Dr Sauter is a consultant for Atossa Genetics, Inc.

## Authors' contributions

WQ conducted total and individual protein assessment on all samples. KZ performed statistical analysis for the study. BK managed and provided quality assurance of the data analyzed. RLR assisted with preliminary data analysis and provided critical review of the manuscript. EPB performed methylation analysis and manuscript review. KFA oversaw methylation analysis and provided manuscript review. ERS initiated the study, oversaw the conduct of the entire project, enrolled participants, and prepared the manuscript for publication. All authors read and approved the final manuscript.

## Pre-publication history

The pre-publication history for this paper can be accessed here:

http://www.biomedcentral.com/1471-2407/12/100/prepub
